# Development and selection of low-level multi-drug resistance over an extended range of sub-inhibitory ciprofloxacin concentrations in *Escherichia coli*

**DOI:** 10.1038/s41598-020-65602-z

**Published:** 2020-05-29

**Authors:** Carly Ching, Muhammad H. Zaman

**Affiliations:** 1 Boston University, Department of Biomedical Engineering, Boston, MA USA; 2 Howard Hughes Medical Institute, Boston University, Boston, MA USA

**Keywords:** Microbiology, Antimicrobials, Bacteria

## Abstract

To better combat bacterial antibiotic resistance, a growing global health threat, it is imperative to understand its drivers and underlying biological mechanisms. One potential driver of antibiotic resistance is exposure to sub-inhibitory concentrations of antibiotics. This occurs in both the environment and clinic, from agricultural contamination to incorrect dosing and usage of poor-quality medicines. To better understand this driver, we tested the effect of a broad range of ciprofloxacin concentrations on antibiotic resistance development in *Escherichia coli*. We observed the emergence of stable, low-level multi-drug resistance that was both time and concentration dependent. Furthermore, we identified a spectrum of single mutations in strains with resistant phenotypes, both previously described and novel. Low-level class-wide resistance, which often goes undetected in the clinic, may allow for bacterial survival and establishment of a reservoir for outbreaks of high-level antibiotic resistant infections.

## Introduction

Antibiotic resistant infections can undermine effective treatments on which we typically rely on. It is thus critical to understand how bacteria develop resistance to better design appropriate interventions. While focus is often given to genetic determinants and mechanisms associated with high-level and clinically-relevant antibiotic resistance during lethal selection^1^, bacteria are also often exposed to lower, sublethal concentrations of antibiotics. This occurs in the environment during agricultural activities and wastewater treatment^2,3^. It also occurs in the clinic due to low-dose prophylactic treatment, incorrect dosing, poor patient adherence and use of poor quality or substandard medicines, which often do not have the stated amount of active pharmaceutical ingredient (API)^4,5^. This prevalent, and often inadvertent, exposure of bacteria to sub-inhibitory levels of antibiotics may serve as an important driver of antibiotic resistance^6^.

When exposed to an antibiotic at levels below the minimum inhibitory concentration (MIC), bacteria survive and are under selective pressure to gain resistance^6^. Recent work has shown that there may be different resistance mechanisms induced by sub-inhibitory antibiotic exposure compared to lethal selection. In *Salmonella enterica*, sub-inhibitory concentrations of streptomycin selected for high-level resistance through multiple small-effect resistance mutations, whereas lethal selection led to specific target mutations^7^. Similarly, resistant mutants from sub-inhibitory fluoroquinolone exposure do not always have causative changes in the target quinolone resistance determining region (QRDR)^8–11^.

We systematically reviewed the literature on sub-inhibitory fluoroquinolone antibiotic exposure and resistance (in preparation &^12^). Briefly, we found that, to date, studies largely have investigated exposure concentrations at or below half the MIC. Many experiments also examined resistance development with passage of bacteria in continually increasing sub-inhibitory concentrations^13–16^. It is less clear what happens upon repeated exposure to a range of constant sub-inhibitory antibiotic concentrations. This is relevant as substandard antibiotics have been found to have a wide distribution of API content^17–19^. Our aim was to determine the role of exposure to different sub-lethal antibiotic concentrations on bacterial antibiotic resistance development. We focused on the fluoroquinolone ciprofloxacin which is used worldwide in both human and animal sectors, and therefore has both clinical and environmental effects^20–23^.

In this study, we investigated the impact of a wide range of sub-inhibitory concentrations of ciprofloxacin, from 0% to 110% of the MIC, on stable antibiotic resistance acquisition in *Escherichia coli*. After exposure, we observed low-level resistance to both ciprofloxacin and other classes of antibiotics in a time- and concentration-dependent manner. We identified a spectrum of single mutations conferring intermediate multi-drug resistance phenotypes. These include both the reported clinically relevant mutation V29G in the efflux regulator *acrR*^24^ as well as mutations I534S in *gyrA* and L75R in the efflux regulator *marR* that are not reported in the literature, to the best of our knowledge.

Overall, our findings add to evidence that low-level antibiotic exposure and low-level resistances prime bacteria for further survival and high-level multi-drug resistance development. We find, specifically, that this can occur at a broad range of sub-inhibitory ciprofloxacin concentrations. Notably, while low-level resistant bacteria have stable genetic changes, they may not be phenotypically classified as clinically resistant based on change in MIC. This has broad health implications, from agriculture to poor quality antibiotic usage, as this may result in strains of bacteria that are pre-disposed to evolve further resistances.

## Results

### Survival decreased sharply with exposure to increasing sub-inhibitory ciprofloxacin concentrations

**Figure 1:**
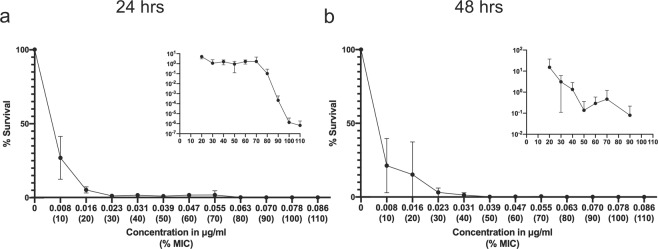
Survival decreased sharply with exposure to increasing sub-inhibitory ciprofloxacin concentrations. Percent survival (relative to no drug treatment) plotted against ciprofloxacin (Cip) concentration (% MIC noted below) after (**a**) 24 hr exposure and (**b**) 48 hr exposure. Error bars represent the standard deviation of the mean of 3 independent experiments. For better resolution of small values, inset shows survival at ≥20% MIC Cip exposure, when survival is less than 20%. The growth observed at 100% and 110% (~50–100 CFU/ml) at 24 hrs was from 1 experiment. Inset is on a log scale, and thus values of 0 are not plotted.

To test the impact of a broad range of sub-inhibitory concentrations of ciprofloxacin API on cell survival, we first determined the MIC of ciprofloxacin for the parental *E. coli* MG1655 strain using a standard broth microdilution. Based on growth, it was found that 0.078 µg/ml was the MIC. Bacteria were then exposed to increasing concentrations of ciprofloxacin, ranging from 0–110% MIC, in 10% increments. The cultures were treated for 48 hours (hrs), with a passage into fresh medium containing the same concentration of ciprofloxacin after 24 hrs of treatment and viable cells were enumerated. Survival of *E. coli* cells decreased sharply after exposure to concentrations greater than 10% MIC, with viable cells typically remaining detectable until about 90% MIC exposure (Fig. 1). This confirmed our defined inhibitory range within 10–20% of the set 100% MIC value.

### Low-level ciprofloxacin resistance occurred after 20–30% MIC exposure

**Figure 2:**
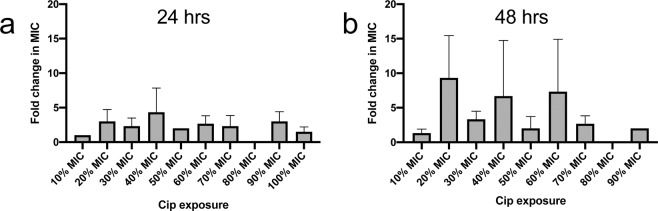
Low-level increases in resistance develop after 20–30% MIC exposure. Fold change increase (relative to drug-free control) in MIC of ciprofloxacin (Cip) plotted against ciprofloxacin concentration treatment (as % MIC) after (**a**) 24 hr exposure and (**b**) 48 hr exposure. Error bars represent the standard deviation of the mean of 3 independent experiments. Experiments with no detectable cells were not included in calculation. A fold change of 1 indicates no change. A value of 0 indicates that there were no detectable cells from the initial treatment. The MIC of the drug-free control matched the MIC of WT parental cells.

The MIC of the exposed cells to ciprofloxacin was determined after a single outgrowth in drug-free media. An increase in MIC relative to the MIC of the drug-free control was observed after bacteria were exposed to 20–90% MIC ciprofloxacin (Fig. 2). Exposure to 10% MIC, despite causing a large reduction in survival (~75%, Fig. 1), resulted in no increase in resistance at both time points. Increases in MIC ranged from ~2–16 fold, with no obvious relationship to MIC concentration directly after antibiotic exposure between three independent lineages.

### Mutant strains demonstrated stable low-level resistance which increased with exposure concentration and time

**Figure 3:**
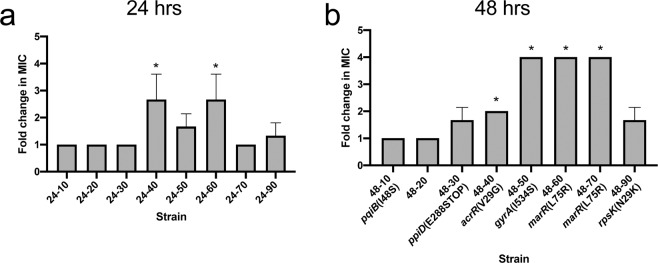
Mutant strains after passage in drug-free medium have low-level resistance, fold change in resistance increases with % MIC. Cells were passaged on drug-free media for 5 days and the MIC to ciprofloxacin was tested. Each strain name (#-#) corresponds to exposure time-ciprofloxacin concentration (% MIC). Fold change (relative to drug-free control) in MIC of ciprofloxacin is plotted against ciprofloxacin treatment concentration (as % MIC) for mutant from (**a**) 24 hr exposure or (**b**) 48 hr exposure. Error bars represent the standard deviation of the mean of 3 replicate measurements. A fold change of 1 indicates no change. Drug-free control matched MIC of parental WT cells and conditions with no growth during purification were not plotted. All non-synonymous genetic changes identified in sequencing are shown below strain name. *P value < 0.05.

To test whether the resistance changes were stable, treated cells were passaged on drug-free media for 5 days. After passage, the increase in MIC stabilized to a maximum of 4-fold, with changes observed after ≥30% MIC exposure (Fig. 3). These strains were named by their time of treatment and % MIC exposure. For example, strain 24–10 was treated for 24hrs with 10% MIC ciprofloxacin. After drug-free transfer, we noted that resistance increased with increased exposure concentration and time. We performed a Wilcoxon ranked sum test to determine whether the mutants’ resistance profile were representative of a non-identical population compared to the drug-free control. We found that increases in MIC for mutants 48-40, 48–50, 48–60 and 48–70 were significant (P values <0.05, Fig. 3), such that they would not be due to technical error or chance. Strong growth defects were observed in strains 24–90 and 48–90 while strains 24–50, 48–30 and 48–50 had a slight growth defect at 37 °C (Fig. S1).

### Ciprofloxacin-selected mutants demonstrated increased multi-drug resistance

**Figure 4:**
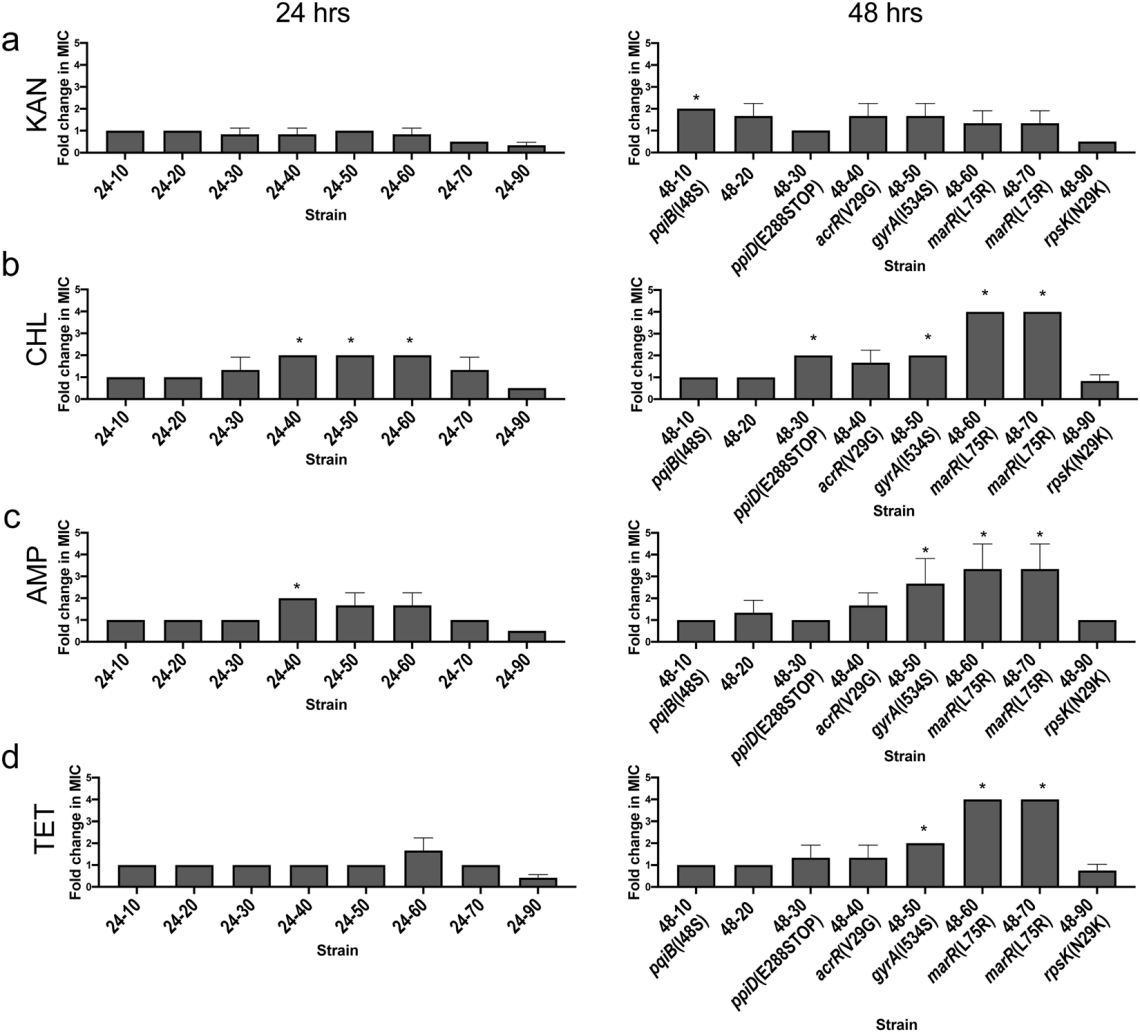
Mutants demonstrate increased multi-drug resistance which increase with concentration and time. MIC of different classes of antibiotic were determined for stable mutants. For 24 and 48 hr exposure mutants, the fold change in MIC relative to drug-free control was measured for 4 different antibiotics, (**a**) kanamycin (KAN, MIC = 16 ug/ml), (**b**) chloramphenicol (CHL, MIC = 8ug/ml) (**c**) ampicillin 1 (AMP, MIC = 8 ug/ml) and (**d**) tetracycline (TET, MIC = 2ug/ml). Error bars represent the standard deviation of the mean of 3 replicate measurements. A fold change of 1 indicates no change. The MIC of the drug-free control matched the MIC of WT parental cells. Conditions with no growth after exposure were not plotted. All nonsynonymous genomic changes identified in sequencing are shown below mutant name. *P value < 0.05.

To investigate whether the resistant mutants had developed multi-drug resistance, we determined the MIC of each stable mutant to antibiotics from 4 different classes (ampicillin, kanamycin, tetracycline, chloramphenicol) (Fig. 4). Interestingly, cross-resistance increased up to 4-fold, similar to increases in resistance to ciprofloxacin. In general, cells from prolonged exposure (48 hrs) had increased resistance to chloramphenicol, ampicillin and tetracycline (P-values < 0.05; Fig. 4b–d, right) but did not show resistance to kanamycin. Cells isolated after 90% MIC exposure (24–90 & 48–90) demonstrated increased sensitivity to all four antibiotics. These data demonstrate that not only does resistance to the primary treatment antibiotic (ciprofloxacin, in this case) develop, but resistance to unrelated classes of antibiotics as well.

### Ciprofloxacin-selected mutant strains had mutations in *gyrA, marR* and *acrR*

To identify genetic changes in the 48-hr exposed mutants, we performed whole genome sequencing (WGS) after sequentially passaging the strains on drug-free media. The results are summarized in Table 1. Notably, we identified only one nonsynonymous mutation in each strain with altered antibiotic resistance. Among the induced mutations, we found a propensity for T:A > G:C transversions (8/9 mutations identified). This mutational signature preference for ciprofloxacin was also observed in *Salmonella typhimurium*^25^. Additionally, we detected four nucleotide changes in our WT parental strain compared to the MG1655 reference genome, listed in Table S1. The sequence mapping analysis was unable to identify possible gene duplications. All mutant strains and the drug-free control also had these same changes.

**Table 1:** Mutations identified in strains from 48 hr sub-inhibitory ciprofloxacin exposure, after passage on drug-free media.

Strain Number	Ref Position (NC_000913)	Type	Ref	Change	Overlapping annotations	Coding region change	Amino acid change	Mean Fold Change in MIC (relative to drug-free control)
48-0	ND							
48-10	1013401	SNP	T	G	Gene: *pqiB*	T143 > G	I48S	1
48-20	ND							1
48-30	462776	SNP	G	T	Gene:*ppiD*	G862 > T	E288to STOP	1.7
	2999250	SNP	A	G	Gene:*ygeR*	T642 > C	T214T	
48-40	485846	SNP	T	G	Gene: *acrR*	T86 > G	V29G	2*
48–50	2337820	SNP	A	C	Gene:*gyrA*	T1601 > G	I534S	4*
48–60	1619343	SNP	T	G	Gene:*marR*	T224 > G	L75R	4*
	3219799	SNP	T	G	Gene:*patA*	T306 > G	V102V	
48–70	1619343	SNP	T	G	Gene:*marR*	T224 > G	L75R	4*
48–90	3442012	SNP	G	T	Gene: *rpsK*	C87 > A	N29K	1.7

Ref = reference, SNP = single nucleotide polymorphism, ND = no differences to WT parental strain.

*P value less than 0.05.

The drug-free control and strain 48–20 had no additional mutations compared to the parental strain. The mutant strain 48-10 had a single nucleotide polymorphism (SNP) leading to a nonsynonymous mutation in the intermembrane transport protein, *pqiB*, which is involved in cell envelope homeostasis. A previous study found that a *pqiB* mutant did not show strong phenotypes to different growth conditions^26^. Mutant 48-30 had a synonymous mutation in the lipoprotein gene *ygeR* and a nonsynonymous mutation in peptidyl-prolyl cis-trans isomerase D, *ppiD*, which has been shown to have a minor role in the maturation of outer membrane proteins^27^. This mutant had a slight growth defect (Fig. S1).

Fluoroquinolones act by binding to DNA gyrase and/or DNA topoisomerase IV to inhibit DNA unwinding and supercoiling^28,29^. Typically, mutations in the fluoroquinolone binding site of DNA gyrase and topoisomerase IV lead to resistance^30^. Strain 48–50, which displayed a 4-fold increase in MIC (P-value = 0.01267), had a mutation in the gyrase gene, *gyrA*, of Isoleucine-534 to Serine. This mutation was predicted to be in the C-terminal domain^31^, outside of the conserved QRDR involved in drug binding^32,33^. The *gyrA*(I534S) mutant had ~2-fold changes in resistance to chloramphenicol, tetracycline and ampicillin (P-values < 0.05, Fig. 4) and a slight growth defect (Fig. S1).

Mutations in genes involved in drug efflux pathways may lead to decreased ciprofloxacin accumulation in the cell and, moreover, decrease accumulation of other unrelated antibiotics. One of the main efflux systems in *E. coli* is the AcrAB-TolC pump which has wide substrate specificity^34,35^. The *acrAB* operon, which encodes the AcrAB-TolC efflux pump, is negatively regulated by the transcriptional repressor AcrR and positively regulated by the transcriptional regulator protein MarA^36,37^. Overexpression of *marA* leads to multi-drug resistance^38^ and MarR is a transcriptional repressor of *marA*^39,40^. In strain 48-40, which had a 2-fold increase in MIC (P-value = 0.01267), Valine-29 was mutated to Glycine in *acrR*. Based on the crystal structure of *acrR*, V29 is predicted to be in the *tetR*-type helix-turn-helix motif^41^(Fig. S4). This mutation was previously identified in high-level levofloxacin-resistant clinical strains and had also been reported in *in vitro*-selected high-level carbapenem resistant isolates^24,42^. The *acrR*(V29G) mutant displayed some changes in resistance to chloramphenicol and ampicillin, but with P values > 0.05 (Fig. 4).

Strains 48–60 and 48–70, which had 4-fold increases in ciprofloxacin MIC (P-value =0.01267), both had the same mutation in *marR* of Leucine-75 to Arginine. Mutant 48–60 also had a synonymous mutation in the aminotransferase *patA*. Leu-75 is predicted to be in the DNA-binding helix-turn-helix regions (Fig. S5), specifically in ﻿H3 (α4, recognition helix) of MarR^43–45^. For *E. coli*, changes in residues 66, 70 and 77–79 of MarR have previously been described in clinical and *in vitro* selected fluoroquinolone resistant isolates carrying other mutations^46–48^, but we could not find mutations in Leu-75 reported within the compiled literature^49^. Interestingly, in a study of *marR* mutations, it was found that most *marR* alleles of clinical isolates had missense amino acid substitutions in *marR* that conferred lower levels of resistance (1.5–4-fold changes in MIC) and lower fitness growth costs than other types of mutations from *in vitro* selection^49^. The *marR*(V29G) mutant had the strongest multi-drug resistant phenotypes for chloramphenicol, ampicillin and tetracycline of ~4-fold (P values < 0.05, Fig. 4). Similarly, the *marR* V84E mutation had a small increase in MIC to ciprofloxacin, ampicillin, chloramphenicol, and tetracycline^48^.

Strain 48–90, had a nonsynonymous mutation in the ribosomal protein *rpsK*(30S ribosomal subunit protein S11) which is essential^50^. Matching its slow growth phenotype (Fig. S1), these cells also have a small colony phenotype which does not revert back to WT physiology after passage on drug-free media (Fig. S2). Cells with the *rpsK* mutation are likely very sick due to a mutated ribosomal protein, which supports its increased sensitivity to other antibiotics (Fig. 4).

Based on these results, we hypothesized that these single point mutations not only confer low-level increases in resistance, but may also allow for growth and further accumulation of new mutations for increased levels of resistance during prolonged selective pressure. Indeed, when cells were passaged for 10 days in corresponding sub-inhibitory concentrations and subsequently passaged on drug-free medium for 5 days, cells became up to 16 to 32-fold more resistant (Fig. S3), likely from accumulation of new resistance mutations.

## Discussion

In this study, we investigated whether antibiotic exposure to a broad range of sub-lethal concentrations led to differences in antibiotic resistance profiles. The results showed low-level multi-drug resistance development after exposure to sub-inhibitory ciprofloxacin concentrations as low as 30% MIC (Figs. 3 & 4). We identified a spectrum of mutations in the multi-drug resistant mutants, including some that have not been previously described (Table 1). Our data suggest that exposure to sub-inhibitory levels of antibiotics selects for first-step mutations that confer stable low-level resistance from both ciprofloxacin target mutations and efflux mechanisms. These may then allow for cells to survive further treatment and gain additional mutations. For drug efflux, the mutation V29G in *acrR* has been reported in clinically resistant isolates^24^ and developed to carbapenems^42^, while the mutation L75R in *marR* has not been implicated in resistance prior to this study. Both mutations are predicted to disrupt DNA-binding to its target promoters (Figs. S4 & S5). We also found that during exposure to lower antibiotic concentrations, two cell envelope maintenance genes, *ppiD* and *pqiB*^26,27^, had nonsynonymous mutations.

Understanding the evolutionary trajectories of bacteria after exposure to antibiotics is critical for developing strategies that prevent emergence of antibiotic resistance from the start^51^. For example, a recent paper discovered that antibiotic tolerance after exposure to a single antibiotic promotes the future development of resistance under combination therapy^52^. While it has been demonstrated that multiple mutations are required to achieve high-level clinical resistance, it has been hypothesized that mutations that result in low-level resistance are the first step to the development of high-level resistance^46,53–55^. Mutations which impact class-wide resistance, such as those observed here in efflux, can allow for survival, which leads to a reservoir of bacteria that are more prone to develop resistance given another selective pressure (including different classes of antibiotics)^56^. However, the identification and contribution of relevant single nucleotide changes can be difficult to analyze. Resistant isolates often have multiple mutations and it can be difficult to determine true differences in a clinical isolate with an unknown parental strain. Moreover, high-level multi-drug resistant clinical isolates are often tested for mutations in distinct gene clusters specific to various antibiotics^57,58^, which may miss single nucleotide changes outside these regions which are related to broader, non-specific mechanisms of resistance. It is also possible that first-step mutations which allow for additional mutations for increased resistance or fitness are lost during selection at high treatment levels. Different types of mutations in *acrR* and *marR* have been observed to evolve at different times under different levels of fluoroquinolones^48,51,59,60^. This suggests that there may be a different trajectory or spectrum of first-step mutations with different initial treatment levels of antibiotics.

This study has important implications for global public health. A key observation of our work is the presence of low-level resistance against several important antibiotics after short term sub-inhibitory ciprofloxacin exposure (Figs. 3 & 4). As described by Hughes and Andersson (2012), it is important to understand the﻿ ﻿significance of individual mutations selected at low-levels of antibiotics, as weak resistance mutations are traditionally disregarded^1^. It is noteworthy that low-level resistances are often disregarded due to statistical significance thresholds or classified as clinically relevant based on CLSI MIC breakpoints. Indeed, based on the current clinical breakpoint for ciprofloxacin (1 µg/ml), clinical resistance was not achieved in our experiment^61^. However, while mutants may not be classified as resistant by clinical standards, low-level resistance should not be disregarded, especially because the underlying mutations may confer broad resistance to other classes of antibiotics (Table 1). This could lead to an undetected, under-reported, and pervasive problem spanning from agricultural settings, in which farmers use intentionally-low doses of antibiotics, to the clinical settings in which there may be a high prevalence of poor-quality medicines which have lower API concentrations than what is stated. Examples include ciprofloxacin eye drops in India, which had API content 16% to 36% below the acceptable range^17^ and ciprofloxacin oral suspensions in Ghana, which only had 67% and 72% API content^18^. A recent study from Laos found that the percent API of antibiotics tested, including ciprofloxacin, spanned a range between 75% and 125%^19^. Substandard antibiotics may also have other issues than low API content such as impurities, poor-quality excipients, or degradation products which all may serve as their own stressors and impact the cells’ response and development of resistance.

Thus, our data demonstrates that in the case of ciprofloxacin, practices and situations that lead to sub-inhibitory antibiotic exposure such as during poor-quality antibiotic usage or agricultural activities, could lead to low-level multi-drug resistance which may go undetectable in clinical settings. We found that increased exposure to the same sub-inhibitory levels led to higher levels of resistance (Fig. S3), further suggesting that these low-level resistances can serve as a gateway to higher levels of resistances and, importantly, class-wide multi-drug resistances^54^. Increased surveillance and reporting of low-level resistances is therefore important to consider. Our data also suggest that changes in drug efflux are a major mechanism upon pressure from sub-lethal ciprofloxacin exposure in *E. coli*. This is an important area for deeper study; concentration-dependent responses are relevant as they underly different anthropic drivers of resistance. This extends beyond changes in resistance genes, as community behavior may also change in response to different concentrations of antibiotics^62^. Overall, antibiotic resistance is a complex process, and should be viewed from a systems perspective, taking into consideration the social drivers, scientific outcomes and cellular mechanisms altogether.

## Methods

### Strains and culture conditions

*E. coli* MG1655 (ATCC 700926) was used for all experiments. All cultures were routinely grown in LB medium and incubated at 37 °C with shaking at 180 rpm for liquid cultures. Ciprofloxacin (MP Biomedical) was added to the medium as indicated.

### Ciprofloxacin treatment and susceptibility and survival measurements

For Ciprofloxacin treatment, saturated liquid cultures were diluted 1:100 in 4 mL of LB broth and grown to exponential phase for 2 hrs (~5 × 10^8^ CFU/ml). Cells were then added at a 1:1000 dilution into 4 mL of LB broth containing 0–110% (10% increments) of the Ciprofloxacin MIC (MIC = 0.039–0.078 µg/ml, top of range used) for 24 hrs. After 24 hrs, 4 µl of cells from each exposure condition was added to 4 mL of fresh media containing the corresponding initial treatment (i.e, cells treated with 10% MIC were sub-cultured into fresh media containing 10% MIC) for another 24 hrs (48 hr total exposure). Cultures directly after exposure were frozen with 50% glycerol. Experiments were performed independently, in triplicate. For longer exposure times (Fig. S3), the same protocol was followed, with transfer to fresh media with the corresponding concentration each day (24 hr time period).

To determine the MIC of cells after 24 and 48 hr drug exposure, cells were outgrown in drug-free media overnight and subsequently used in a standard broth microdilution MIC in a 96-well plate using LB media^63^.

To determine survival after 24 and 48 hr drug exposure, bacteria were diluted and plated on LB agar plates. After overnight incubation at 37 °C in a static incubator, colony forming units (CFU) were enumerated. Survival was calculated by dividing the CFU/ml of the sample by the CFU/ml of the drug-free control.

### Drug-free passaging & growth measurements

Mutant strains were inoculated onto LB agar using an inoculation loop. To ensure the MIC of the population, individual colonies were tested for growth in the new MIC. A resistant isolate for each concentration and time combination was transferred daily on drug-free media for 5 days. A single colony was cultured overnight in drug-free media and frozen with 50% glycerol. An MIC for these mutants was performed for Ciprofloxacin, Ampicillin (Sigma-Aldrich), Chloramphenicol (Sigma-Aldrich), Tetracycline (Sigma-Aldrich) and Kanamycin (Sigma-Aldrich).

To monitor growth, O.D. 600 was measured every 5 minutes using a Biotek plate reader with shaking in between each measurement. Wells were seeded with exponential phase cells such that the starting O.D. 600 of each well was ~0.08–0.09. To avoid condensation at 37 °C we made the plate cover hydrophilic as previously described^64^.

### Statistical analysis

All fold changes in MIC were expressed relative to the drug-free control, which matched the MIC of WT parental cells in all experiments. MIC measurements do not follow normal distributions assumed for parametric statistical tests^65^. Therefore, we performed a one-tailed Wilcoxon ranked sum test to determine whether the MIC of stains after treatment were statistically significantly higher compared to the MIC of the drug-free control sample.

### Whole genome sequencing

DNA was extracted using the Qiagen Blood and Tissue Extraction Kit according to manufacturer’s protocol. Paired-end whole genome sequencing was performed at the Broad Institute (Cambridge, MA) using the Illumina Novaseq 6000 platform and the Illumina Novaseq S4 flow cells. The Illumina Nextera XT DNA library prep kit was used to prepare DNA libraries according to the manufacturer’s protocol using ~60 ng of DNA at 2 ng/uL. The target insert size was 300 bp–1.5 kb. Data processing was performed using CLC Genomics Workbench (Qiagen). Sequencing reads from unaligned BAM files, which is a generic format for storing large nucleotide sequence alignments, were aligned to the reference *E. coli* MG1655 genome FASTQ file (NC_ 000913) downloaded from NCBI. Detection of SNPs and insertions and deletions was performed using CLC Genomics Workbench (Qiagen). The average depth of sequencing coverage for each sample is provided in Table S2. A negative control (water) as well as two types of positive controls (mixed microbial sample, and a bacterial isolate control from ZymoBIOMICS) were run in parallel. These controls were included in extraction, library construction and through sequencing to confirm data quality. The sequencing reads were deposited in the NCBI SRA database under BioProject accession number PRJNA611936.

## Supplementary information

Supplemental information.
