# Supplementary material for: Development and selection of low-level multi-drug resistance over an extended range of sub-inhibitory ciprofloxacin concentrations in Escherichia coli
Source: Sci Rep. 2020 May 29;10:8754. doi: 10.1038/s41598-020-65602-z (PMC7260183; doi:10.1038/s41598-020-65602-z)
Supplement: Supplementary file 1 — Supplemental information. [file 41598_2020_65602_MOESM1_ESM.pdf]

Development and selection of low-level multi-drug resistance over an extended range of sub-inhibitory ciprofloxacin concentrations in *Escherichia coli*

Carly Ching[1] & Muhammad H. Zaman[1,2]

1. Boston University, Department of Biomedical Engineering, Boston, MA, USA
2. Howard Hughes Medical Institute, Boston University, Boston, MA USA

Table S1. Nucleotide of parental wildtype strain compared to reference genome

Strain Number	Reference Position (NC_000913)	Type	Referen ce	Change	Overlapping annotations	Coding region change	Amino acid change
WT	257908	SNP	G	A	Mobile element	G>A	
	2173361	Del	CC	-	Gene: *gatC*	917_918del	Gly306fs
	3560456	Ins	-	G	Gene: *glpR*	150_151insC	Ala51fs
	4296381	Ins	-	CG	Repeat region: REP321j		

SNP = single nucleotide polymorphism, Del= deletion, Ins= insertion, fs=frameshift

Table S2. Depth of sequencing coverage for each sample

Strain Number	Nonsynonymous changes	Avg. Fold Coverage (reads/bp)
WT	Table S1	723
48-0	ND	674
48-10	*pqiB*(I48S)	452
48-20	ND	985
48-30	*ppiD(*E288toSTOP)	646
48-40	*acrR* (V29G)	782
48-50	*gyrA* (I534S)	748
48-60	*marR*(L75R)	447
48-70	*marR*(L75R)	1031
48-90	*rpsK*(N29K)	192

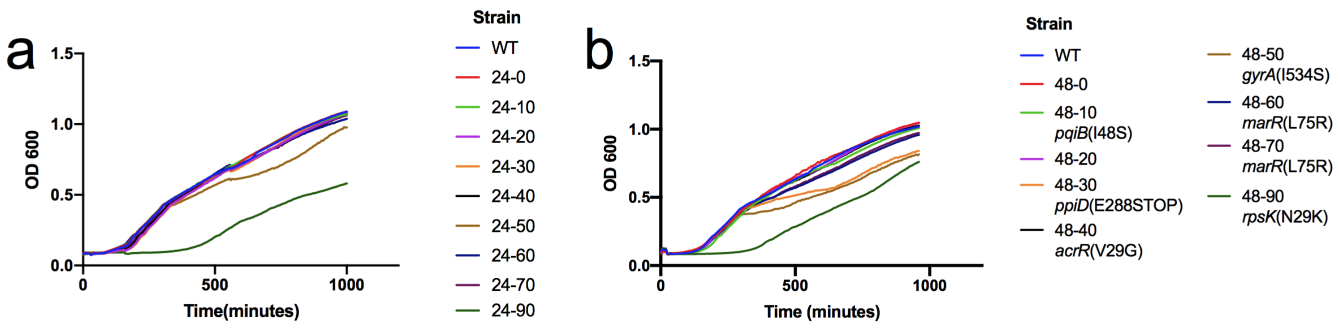

Figure S1. Most strains display WT growth in the absence of antibiotic. Growth curve (OD 600 vs Time) for strains from (a) 24 hr and (b) 48 hr ciprofloxacin exposure at 37 °C. Graph is representative of 3 replicates.

a

20% MIC exposure – 48 hrs

b

90% MIC exposure – 48 hrs

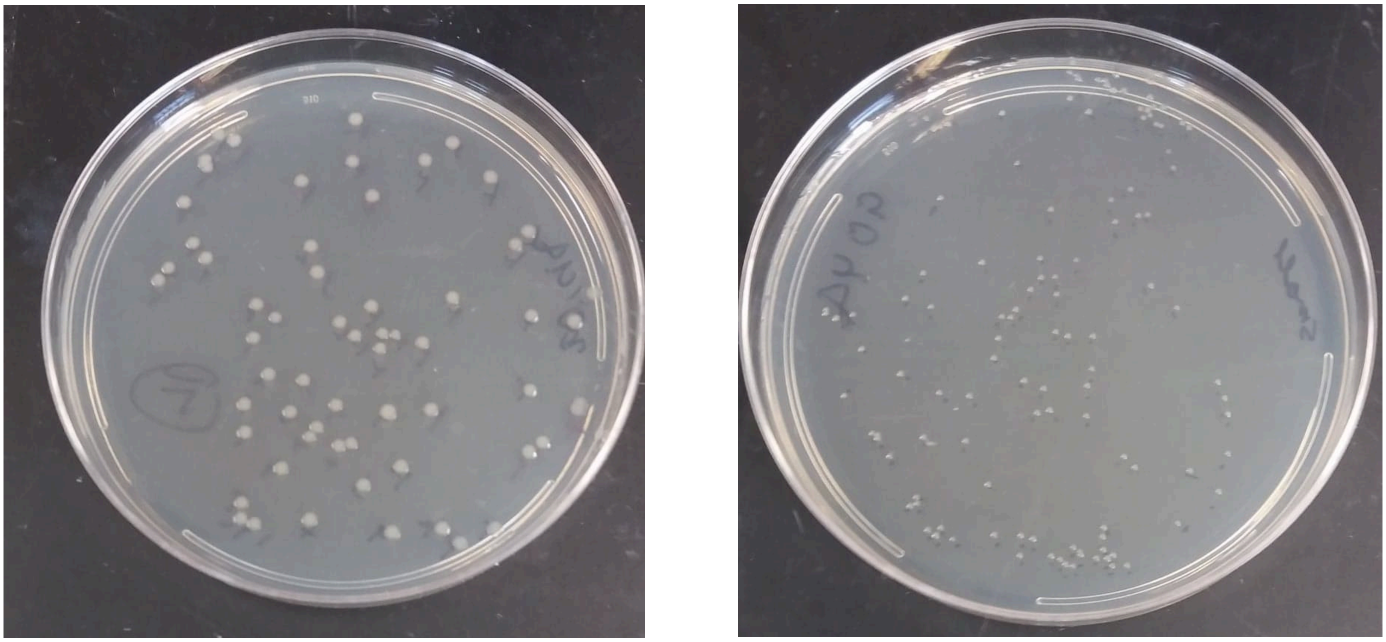

Figure S2. Cells after exposure with 90% MIC ciprofloxacin (replicate 2) grown overnight display a small colony phenotype (b) compared with treatment at lower concentrations which appear WT (a) on drug-free medium.

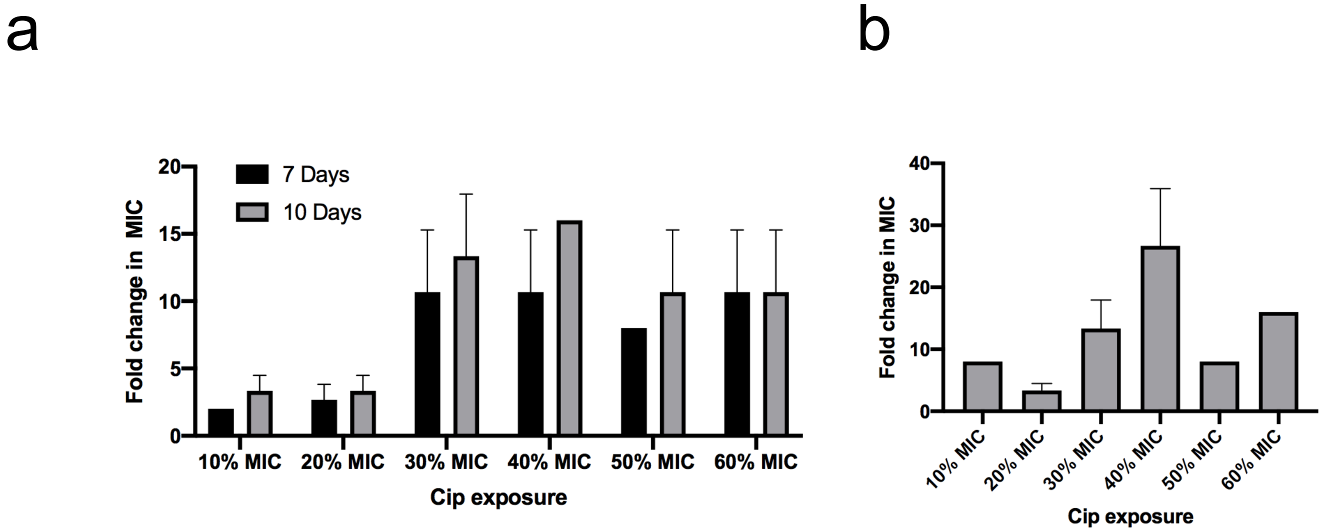

Figure S3. Increased exposure time to sub-inhibitory ciprofloxacin concentrations leads to increased resistance. (a) Fold change (relative to drug-free control cells) in MIC of ciprofloxacin plotted against ciprofloxacin treatment concentration (as % MIC) after 7-day (black bar) and 10-day (grey bar) exposure. Cells were transferred to fresh media and ciprofloxacin each day. (b) Cells from 10-day exposure were purified on drug free-media for 5 days and MIC to ciprofloxacin was measured. Error bars represent the standard deviation of the mean of 3 replicate measurements. Drug-free controls (0%) maintained the same MIC values as parental WT cells. All changes had P values < 0.05.

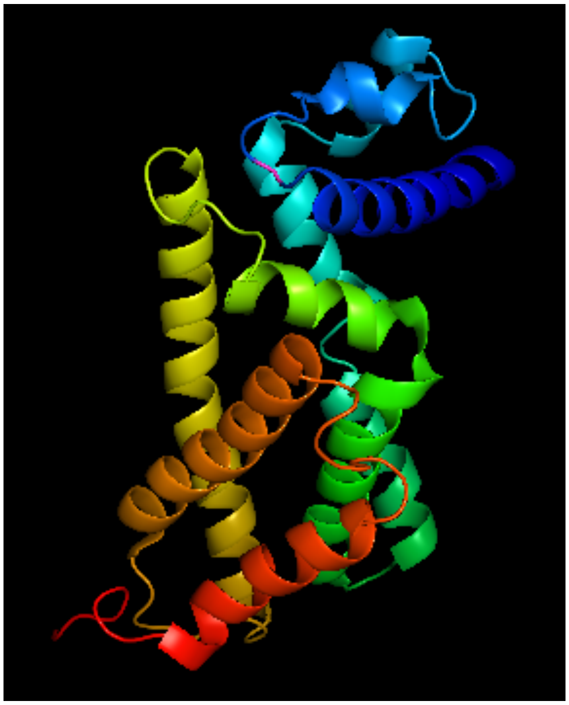

Figure S4. Structure of *E. coli acrR* (PDB 2QOP)(1) created with PyMol. The protein is displayed in cartoon style and colored by chainbows (blue to red from its N-terminus to its C-terminus). Valine 29 is highlighted in magenta. The experimental method for structure generation was X-ray diffraction with a resolution of 2.55 angstroms.

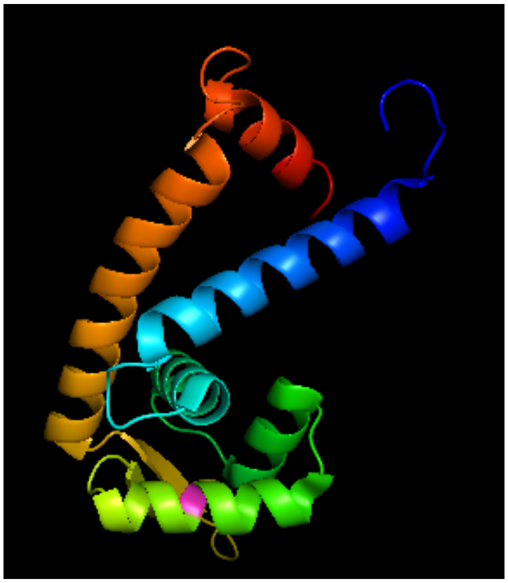

Figure S5. Structure of *E. coli marR* (PDB 1JGS)(2) created with PyMol. The protein is displayed in cartoon style and colored by chainbows (blue to red from its N-terminus to its C-terminus). Leucine 75 is highlighted in magenta. The experimental method for structure generation was X-ray diffraction with a resolution of 2.3 Angstroms.

References:

1. Li M, Gu R, Su CC, Routh MD, Harris KC, Jewell ES, McDermott G, Yu EW. 2007. Crystal Structure of the Transcriptional Regulator AcrR from Escherichia coli. J Mol Biol 374:591–603.
2. Alekshun MN, Levy SB, Mealy TR, Seaton BA, Head JF. 2001. The crystal structure of MarR, a regulator of multiple antibiotic resistance, at 2.3 Å resolution. Nat Struct Biol 8:710–714.
